# Blood-Serum Therapy and Antitoxines

**Published:** 1895-12

**Authors:** 


BLOOD-SERUM THERAPY AND ANTITOXINES. By Geo. E. Krieger, M. D., Chicago, E. H. Colegrove & Co., Publishers. Pp. 68, with illustrations.

This book will be received with considerable favor just now on account of the interest felt by the profession, and the general public as well, in the subject of antitoxines. It furnishes interesting information respecting the treatment of diphtheria and tetanus, gives a clear account of the status of serum therapy, and the methods of producing toxines and antitoxines, and their sources, and is a fair exposition of the subjects under discussion.

